# Development of New Tools to Detect Colistin-Resistance among Enterobacteriaceae Strains

**DOI:** 10.1155/2018/3095249

**Published:** 2018-12-05

**Authors:** Lucie Bardet, Jean-Marc Rolain

**Affiliations:** Aix-Marseille Université, IRD, AP-HM, MEPHI, IHU-Méditerranée Infection, Marseille, France

## Abstract

The recent discovery of the plasmid-mediated *mcr-1* gene conferring resistance to colistin is of clinical concern. The worldwide screening of this resistance mechanism among samples of different origins has highlighted the urgent need to improve the detection of colistin-resistant isolates in clinical microbiology laboratories. Currently, phenotypic methods used to detect colistin resistance are not necessarily suitable as the main characteristic of the *mcr* genes is the low level of resistance that they confer, close to the clinical breakpoint recommended jointly by the CLSI and EUCAST expert systems (*S *≤* *2* *mg/L and *R *>* *2* *mg/L). In this context, susceptibility testing recommendations for polymyxins have evolved and are becoming difficult to implement in routine laboratory work. The large number of mechanisms and genes involved in colistin resistance limits the access to rapid detection by molecular biology. It is therefore necessary to implement well-defined protocols using specific tools to detect all colistin-resistant bacteria. This review aims to summarize the current clinical microbiology diagnosis techniques and their ability to detect all colistin resistance mechanisms and describe new tools specifically developed to assess plasmid-mediated colistin resistance. Phenotyping, susceptibility testing, and genotyping methods are presented, including an update on recent studies related to the development of specific techniques.

## 1. Introduction

**Figure 1:**
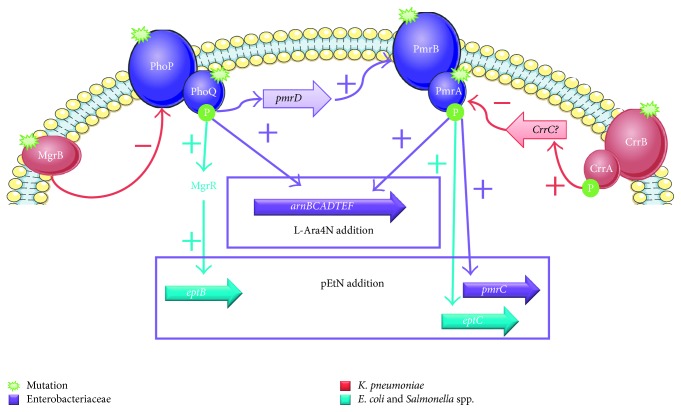
Molecular mechanisms of acquired resistance to polymyxins. L-Ara4N: 4-amino-4-arabinose; pEtN: phosphoethanolamine.

Multidrug-resistant (MDR) bacteria are of a global concern, notably with the description of carbapenemase-producing Enterobacteriaceae [1]. Colistin is an old antibiotic that regained popularity as a last resort treatment to face the worldwide emergence of these pathogens [2]. Colistin is a polycationic and bactericidal drug that targets the lipid A moiety of lipopolysaccharide (LPS), moving its cationic charges, leading to cell wall lysis and bacterial death [3]. The increasing use of colistin has led to emerging resistance, a phenomenon that represents a clinical source of worry [4]. Enterobacteriaceae are Gram-negative bacteria that are often described as the pathogens responsible for human infectious diseases, particularly the *Escherichia coli* and *Klebsiella pneumoniae* species. Until recently, all mechanisms described were of chromosomal origin, mostly mediated by the two-component systems PmrAB and PhoPQ, leading to the addition of positively charged carbohydrates on the negatively charged lipid A (Figure 1), a phosphoethanolamine by a phosphoethanolamine transferase or a 4-amino-4-arabinose by surexpression of *arnBCADTEF* operon, leading to the loss of polymyxin affinity for the LPS [5]. In November 2015, Liu et al. reported the first plasmid-mediated gene which they named *mcr-1* [6], which encodes for a phosphoethanolamine transferase, and this was followed by the description of variants (*mcr-1.2*, *mcr-1.3*,…) and the genes *mcr-2*, *mcr-3*, *mcr-4*, *mcr-5*,* mcr-6*, *mcr-7*, and *mcr-8* [7–15]. This recent discovery raised concern about the increase and spread of resistance among the Enterobacteriaceae [16] and led to new recommendations for laboratory diagnosis and clinicians [17]. Specifically, the majority of these *mcr-1* strains exhibited a low minimal inhibitory concentration (MIC) of colistin, around 4 *µ*g/ml [6], which is close to the MIC breakpoint according to the EUCAST guidelines (susceptibility* *≤* *2* µ*g/ml and resistance* *>* *2* µ*g/ml) (http://www.eucast.org). Moreover, several studies have reported the detection of the *mcr-1* gene in carbapenemase-producing Enterobacteriaceae strains, describing coproduction with other plasmid-mediated genes (*bla*_*NDM-1*_, *bla*_*NDM-5*_, *bla*_*NDM-9*_, *bla*_*KPC-2*_, *bla*_*KPC-3*_, *bla*_*OXA-48*_, and *bla*_*OXA-181*_) [18–24].

The emergence of antibiotic resistance of clinical interest usually conduces to the development of new tools in clinical microbiology laboratories [25]. Currently, the detection of carbapenemase-producing bacteria is well determined, combining specific culture media, phenotyping testing, antibiotic susceptibility testing, and molecular biology [26–28]. As colistin resistance is a recent global phenomenon, the implementation of rapid and reliable screening tools to detect and analyze colistin-resistant pathogens in such a way as to isolate the patient and adapt the treatment is a necessary approach [29]. Moreover, heteroresistance to colistin is a common phenomenon that is widely underestimated, requiring specific methods [30–32]. Here, we propose an overview of all the screening and analysis methods developed to assess colistin resistance among bacterial pathogens causing infectious diseases in hospitalized patients. This review summarizes the current clinical microbiology diagnosis techniques and their ability to detect all colistin resistance mechanisms, and describes new tools specifically developed to assess plasmid-mediated colistin resistance [33].

Phenotyping, susceptibility testing, and genotyping methods are presented, including an update on recent studies related to the development of specific techniques.

## 2. Phenotypic Detection Methods

### 2.1. Selective Culture Media

Culture remains the benchmark method for isolating pathogens within clinical samples, and selective media are continuously developed to isolate specific bacteria [25]. Until recently, there was no specific culture medium for the detection of colistin-resistant strains, and current polymyxin-containing culture media were not able to detect low-level resistant strains because the concentrations of polymyxin in their composition are too high or because they contain other antimicrobial drugs [34–61] (Table 1). Therefore, some in-house media have been developed for colistin-resistant strain screening studies, including strains carrying the *mcr* genes (Supplementary Table S1). These selective culture media were developed by adding low concentrations of colistin (2 or 4 mg/L) to LB nonselective agar or a MacConkey medium, which is selective of Gram-positive contaminants [62, 63]. The chromogenic and nonselective CHROMagar Orientation medium (Biomérieux, Marcy l'Étoile, France) was also used with 4 mg/ml of colistin [64]. They were used in studies to detect the growth of colistin-resistant isolates by directly culturing samples [65–67] or following an enrichment step [68] which could also be selective with the addition of 2 mg/L of colistin to the broth medium [62, 64, 69]. Other anti-infective drugs could be added to avoid contaminants: vancomycin for Gram-positive contaminants [64, 66, 68] and/or amphotericin B for fungal pathogens [67, 68]. For some other studies, such media were developed to screen colistin resistance in bacterial isolates by subculturing them on agar with 2 mg/L of colistin: MH agar [9], COS medium [70], or MacConkey medium [65]. Wong et al. named their medium MHC1 for Mueller–Hinton colistin 1 [71]. Lastly, the selective CNA medium (colistin and nalidixic acid-containing agar), containing 10 mg/L of colistin, could detect *mcr-1*-positive isolates, one *E. coli* [72] and one *K. pneumoniae* [73], and was also used with CLED (cysteine lactose electrolyte deficient) medium (BioMérieux, Marcy l'Étoile, France) for screening samples that had or had not been precultured on Trypticase Soy Broth  ±2 mg/L of colistin [74].

More specifically, the SuperPolymyxin medium (Elitech Microbio, Signes, France) was developed and intended to specifically detect colistin-resistant strains, including those with a low MIC of colistin and harboring the *mcr-1* gene [75]. The SuperPolymyxin medium has the advantage of facilitating the visualization of *E. coli* strains because it is composed of EMB agar, meaning that they exhibit a metallic green reflect. Its specificity is enabled by 3.5* µ*g/ml of colistin, 10* µ*g/ml of daptomycin, and 5* µ*g/ml of amphotericin B in its composition.

The CHROMagar COL-APSE medium was also developed to detect colistin-resistant strains and was compared to the SuperPolymyxin [76]. Its composition is not precisely described, based on commercial CHROMagar compounds containing colistin sulfate and oxazolidonone antibiotics. The CHROMagar COL-APSE medium presents the advantage to be chromogenic, with the capacity to differentiate colistin-resistant nonfermentative Gram-negative strains as well as Enterobacteriaceae.

The LBJMR medium was also developed to detect all the colistin-resistant bacteria, including those harboring *mcr-1* genes [77]. The LBJMR medium presents the advantage of being versatile, combining colistin-resistant and vancomycin-resistant bacteria screening tools, conferred by 4* µ*g/ml of colistin sulfate and 50* µ*g/ml of vancomycin. In particular, the LBJMR medium can be used to detect vancomycin-resistant enterococci (VRE), which represents another emerging field of clinical concern. Both colistin-resistant Enterobacteriaceae and VRE strains are easy to detect on the LBJMR medium with the presence of bromocresol purple and glucose: fermentative strains exhibit yellow colonies on a purple agar. Lastly, it can be used to specifically detect pathogens that are often diagnosed in cystic fibrosis patient samples.

The sensitivities of these three media were excellent to detect colistin-resistant strains.

### 2.2. Qualitative Detection of Colistin Resistance with Phenotypic Tests

#### 2.2.1. Rapid NP Polymyxin Test for Enterobacteriaceae

The rapid polymyxin NP test (Elitech, Signes, France) is based on a simple pH test, and detection of colistin resistance is obtained by a color change within 2 hours [78, 79]. The test was evaluated on 200 isolates of Enterobacteriaceae and can be used directly on blood samples [80]. A recent review proposed a diagnosis plan integrating this phenotypic test to confirm colistin resistance of Enterobacteriaceae strains after their growth on a selective medium [29], and its reliability is discussed in several studies [81, 82]. Compared to the broth microdilution (BMD) susceptibility testing method, agreements were excellent to detect *mcr-1* and *mcr-2* strains [83, 84]. The rapid polymyxin test has a good sensitivity to detect *Hafnia* sp. colistin-resistant isolates [79] but failed to detect *Enterobacter* sp. isolates, surely due to their heteroresistance to colistin [85]. This test has to be evaluated with nonfermentative colistin-resistant strains, such as *Acinetobacter baumannii* and *Pseudomonas aeruginosa*.

#### 2.2.2. Micromax Assay for *A. baumannii*

The Micromax assay (Halotech DNA SL, Madrid, Spain) is based on the detection of DNA fragmentation and cell wall damage in the presence of colistin [86]. Bacteria are incubated for 60 min with 0.5* µ*g/ml of colistin, trapped in a microgel, and then incubated with a lysis solution to remove weakened cell walls. The presence of DNA fragments is detected after staining by SYBR Gold fluorochrome and observed by fluorescence microscopy. Resistance corresponds to ≤11% of bacteria with cell wall damage. This method is rapid (3 h 30 min) and showed an excellent sensitivity for the detection of colistin resistance on the 70 *A. baumannii* tested isolates (50 susceptible and 20 resistant), but it is not specific for determining the resistance type.

### 2.3. Specific Phenotypic Screening Methods for the Detection of *MCR-1*

#### 2.3.1. Matrix-Assisted Laser Desorption-Ionization Time-of-Flight Mass Spectrometry (MALDI-TOF MS)

The detection of polymyxin-resistant bacteria by MALDI-TOF is a promising and costless approach, as the majority of clinical microbiology laboratories own the required equipment to routinely identify clinical isolates [87]. Currently, the use of MALDI-TOF for detecting the carbapenemase-producing bacteria is described, with the detection of carbapenem hydrolysis [88–90]. As a specific peak was described for lipid A at 1796.2 m/z [91], the MALDI-TOF could be used for the detection of lipid A modifications [92]. Very recently, the MALDIxin test was developed for *E. coli* strains, based on the detection of phosphoethanolamine addition on lipid A, and could specifically detect the *mcr*-positive isolates [93]. Indeed, an additional peak at 1919.2 m/z was observed for all polymyxin-resistant strains, and a second additional peak at 1821.2 m/z was observed for all the *mcr*-positives. The MALDIxin test could detect polymyxin-resistant *E. coli* and also differentiate the chromosome- and plasmid-encoded resistance in 15 minutes, and should be evaluated on other species for which phosphoethanolamine addition is involved in polymyxin resistance.

#### 2.3.2. Inhibition of *MCR-1* Activity

Several studies on the structure of the catalytic domain of the *MCR-1* protein have demonstrated that the phosphoethanolamine transferase is a zinc metalloprotein [94–96], and that zinc deprivation could reduce the colistin MIC in *E. coli* isolates [97]. Screening tests were developed to specifically detect *MCR-1*, based on the difference of colistin susceptibility obtained in the presence or absence of chelators of zinc ion.

The colistin-MAC test consists of the addition of dipicolinic acid (DPA) in the BMD method, leading to a colistin MIC reduction of ≥ 8-fold in case of *MCR-1*-positive strain [98]. 74 colistin-resistant Enterobacteriaceae strains were tested, and 59 of the 61 strains carrying *mcr-1*-like genes were detected by the colistin-MAC test, while the 13 *mcr*-negative strains exhibited discrepancy in results (increase, maintain, or slow decrease) giving a sensitivity of 96.7% and specificity of 100%. Interestingly, the two *mcr-1* strains that were negative with the colistin-MAC test were *K. pneumoniae* strains.

More recently, four assays were tested, based on inhibition by EDTA [99]. The specific detection of *MCR-1* was assessed with the following tests: combined-disk method with diameter differences ≥3 mm, BMD with a reduction of colistin MIC of  ≥4-fold, modified rapid polymyxin NP test with the absence of color change, and the alteration of zeta potential *R*_ZP_ ≥ 2.5. These assays were performed on 109 Enterobacteriaceae including 59 *mcr-1*-positive *E. coli* and one *mcr-1*-positive *K. pneumoniae*. The modified rapid NP test and zeta potential methods showed excellent sensitivity and specificity and could be inexpensive and simple methods to detect the presence of the *mcr-1* gene.

These tests should be performed on other species harboring the *mcr-1* gene, in particular *K. pneumoniae*, and also on strains harboring other *mcr* genes, to validate their ability.

## 3. New Recommendations on Polymyxins Susceptibility Testing

Polymyxin susceptibility testing is challenging, as these large and cationic molecules poorly diffuse into the reference cation-enriched Mueller-Hinton (MH2) agar, giving discrepant results, and much more since the description of the *mcr* genes that confer low MICs. Moreover, even in MH2 broth medium, the concentration of cation could largely influence the polymyxin MIC results [64], notably by interacting with the acquired resistance mechanisms of the tested isolates. Defining a reference method for colistin susceptibility testing is a priority, along with the increasing use of polymyxin as last-line therapies.

### 3.1. Reference Method

Broth microdilution (BMD) is the only approved method for colistin MIC determination by both the European Committee on Antibiotic Susceptibility Testing (EUCAST) and the Clinical and Laboratory Standards Institute (CLSI) [100, 101]. BMD has to be performed with colistin sulfate in untreated polystyrene plates without addition of any surfactant (polysorbate 80) (Table 2). The Mueller–Hinton broth medium has to be cation-adjusted, with a final composition of 20–25 mg/L of calcium and 10–12.5 mg/L of magnesium [102]. EUCAST and CLSI joined their recommendations on the polymyxin breakpoint for MIC of Enterobacteriaceae*, P. aeruginosa* and *Acinetobacter* spp. isolates: susceptible *(S)* if* *≤2* µ*g/ml and resistant *(R)* if* * >2 *µ*g/ml [100, 103]. In 2017, EUCAST added a new quality control (QC) strain that has to be used to control the performances of a colistin susceptibility method: *E. coli* NCTC 18853 that harbors the *mcr-1* gene, in addition to *E. coli* ATCC 25922 and *P. aeruginosa* ATCC 27853 [104] (Table 2).

Dilution methods consist of adding colistin to the culture medium in such a way as to obtain twofold dilutions and are prepared according to the CLSI guide M07-A10 [101] and ISO 20776-1 standard (International Standard Organization). Broth macrodilution is performed in tubes when reference broth microdilution (BMD) is performed in 96-well trays. Only colistin sulfate can be used and particular care is required, as the powder is expressed in IU/mg, meaning that the concentrations need to be adjusted according to the CLSI M100 and the manufacturer's instructions [103]. The antibiotic is suspended in sterile water and then diluted in MH2 broth medium before its distribution into 96-well trays. The final bacterial inoculum is 5 × 10^5^ CFU/ml (colony-forming unit) or 5 × 10^4^ CFU/well for the BMD method, prepared using the 0.5 McFarland standard (corresponding to approximately 1 to 2 × 10^8^ CFU/ml) [101]. Trays are then incubated at 35 ± 1°C for 18 ± 2 hours [100, 102]. Results are read visually or with a spectrophotometer.

Broth microdilution is a time-consuming and fastidious way to assess MIC in clinical routines [105, 106]. Many errors can occur, such as an incorrect colistin concentration or dilution. This technique is not well suited to clinical microbiology routines and needs to be automated. Moreover, this method exhibits limitations for assessing heteroresistance. Indeed, the presence of resistant subpopulations can give uninterpretable results due to the presence of skipped wells and has been described for the *Enterobacter* species, as presented in a study of 114 *Enterobacter cloacae* [107]. Population analysis profiling is recommended to confirm heteroresistance [108]. For now, heteroresistance to polymyxins is not correlated with the presence of *mcr* genes.

### 3.2. Comparative Evaluations of Polymyxin Susceptibility Testing Methods

Evaluating antimicrobial susceptibility testing (AST) methods is performed using a comparison with the reference method, as per the ISO 20776-2 standard [109]: a categorical agreement (CA) is obtained when the strain is in the same clinical category (R, I, S), and an essential agreement (EA) is obtained when the MIC is within plus or minus one doubling dilution from the reference MIC. A very major error (VME) corresponds to a false susceptibility result and is calculated using the resistant strains tested, and a major error (ME), in the case of false resistance, is calculated on the number of susceptible strains. Finally, a minor error (MiE) occurs when a strain is classified as Intermediate *(I)* instead of *S* or *R*, or *S* or *R* instead of *I*. A reliable method will obtain the following scores: CA ≥ 90%, EA ≥ 90%, VME ≤ 3%, and ME ≤ 3% [109]. The results of all the comparative studies performed on colistin susceptibility testing are summarized in Table 3 (in Table S2 for polymyxin B). MIC50 and MIC90 correspond to the MIC that inhibits 50 or 90%, respectively, of the tested strains of the same species.

The surfactant polysorbate 80 was previously added to trays to limit polymyxin adherence to polystyrene and is not yet recommended; however, it could induce VME and *mcr* strains might not be detected [31, 110–112]. Albur et al. demonstrated that the polystyrene trays used also have an influence: using tissue-culture-coated round-bottom trays gave a 5.3-fold increase in MIC values compared to noncoated V-bottom trays [113], for the material used [106] (Table S3). A very recent study compared polystyrene coated trays to glass coated trays and also showed very few differences (Table 3) [114].

Until 2013, many comparative studies used agar dilution (AD) as the reference method for polymyxins susceptibility testing, a method that differs from the BMD only because the polymyxins are added to a solid MH2 medium [31, 32, 115–126]. Compared to BMD, agar dilution generally gave concordant results for colistin and polymyxin B [31, 110, 127, 128]. VMEs were very uncommon with AD, and this pointed to the AD's potential role in screening, as it presents the advantage to test several strains on the same plates [117, 129]. A recent study compared agar dilution to broth macro- and microdilution on 8 strains and concluded that agar dilution was the most reproducible method, with an excellent distribution of colistin in agar, but that colistin-containing agar plates could be only stored for 7 days [130].

Diffusion methods based on the antibiotic diffusion in agar, whether with the Kirby–Bauer disk diffusion [131] or with the gradient strips, are not reliable for polymyxin testing and should not be used as a large number of studies have obtained high rates of VMEs or poor EA [32, 120, 125, 128, 132–135]. Some studies showed good results but contained only susceptible strains [136–138]. The influence of MH2 agar composition was assessed: agreement was not affected with agar dilution, but important differences were highlighted with Etest (BioMérieux, Marcy l'Étoile, France) [31, 139]. The advantage of the agar diffusion method is the detection of heteroresistance: colonies present within the inhibition zone correspond to resistant subpopulations [140]. One study compared disc diffusion to Etest method, and a large rate of minor errors occurred [141]. The ColiSpot test consists of replacing the disk of colistin by a drop of a calibrated colistin solution (8* µ*g/ml). Colistin resistance is revealed in the absence of the inhibition zone. This technique was evaluated with 89 colistin-resistant and 52 colistin-susceptible strains and was developed for veterinarian laboratories [142].

### 3.3. Commercial Devices Based on Broth Microdilution Reference Method

Several commercial devices based on BMD reference methods were developed to easily assess the reference method by offering ready-to-use systems. Their advantage is the elimination of critical preparation steps of MH2 medium and antibiotic dilutions. These systems were used to detect *mcr-1* strains and were evaluated by EUCAST, giving correct results, with essential agreement ranging from 82% to 96%, and few MEs or VMEs (http://www.eucast.org/ast_of_bacteria/warnings/) [143].

#### 3.3.1. UMIC Colistine (Biocentric, Bandol, France)

UMIC colistine consists of unitary tests composed of 12-well polystyrene strips, one for growth control and 11 containing dehydrated colistin, with concentrations ranging from 0.06 to 64* µ*g/ml, provided with unitary MH2 tubes. Inoculation is performed simply, after diluting the 0.5 McFarland suspension by 200-fold into the MH2 tubes, by distributing 100 *µ*L of this diluted suspension into the 12 wells of the strip, leading to the rehydration of the antibiotic. The strips are then incubated at 35 ± 1°C using the UMIC box to avoid any desiccation. One comparative evaluation on 71 *A. baumannii* isolates and one on 92 Gram-negative isolates including 76 Enterobacteriaceae highlighted the reliability of the UMIC colistine kit [144, 145].

#### 3.3.2. MIC Strip Colistin (MERLIN Diagnostika Bornheim-Hersel, Germany)

MIC Strip Colistin also consists in unitary 12-well strips with concentrations of dehydrated colistin ranging from 0.06 to 64 *µ*g/mL, and Micronaut-S is a panel composed of different antibiotics on standard 96-well trays. Those systems can be automated with Micronaut ASTroID that concomitantly performs dilution for antimicrobial susceptibility testing (AST) and deposits on the MALDI-TOF target, simultaneously identifying the same colony being tested.

#### 3.3.3. Sensitest Colistin (Liofilchem, Roseto degli Abruzzi, Italy)

It consists of a compact panel of 4 tests containing 7 twofold dilutions of dehydrated colistin (0.25–16* µ*g/ml). It showed excellent correlation with BMD when tested on 353 isolates, including 259 Enterobacteriaceae, 83 harboring the *mcr-1* gene [146]. Recently, a combined Sentitest colistin/piperacillin-tazobactam was developed, with the same design, providing a unitary test for testing both antibiotics, with colistin concentrations ranging from 0.008 to 128* µ*g/ml.

#### 3.3.4. The Sensititre System (Thermo Fisher Scientific, Waltham, MA, USA)

It presents different antibiotics on 96-well trays with a customizable plate layout. Inoculation, incubation, and reading (based on fluorescence) steps can be automated. Chew et al. [147] recently evaluated a Sensititre GNX3F panel containing both polymyxin B and colistin (0.25–4 mg/L) and presented a sensitivity of 95.2% and 100% in detecting the 21 *mcr-1* isolates tested, respectively.

### 3.4. Automated Systems

Automated systems were developed to shorten result timeframes by increasing sensitivity, and also to avoid manipulation bias [148], with incubation and real-time reading. However, by combining several antibiotics, the number of concentrations tested is limited, and they cannot give a real MIC (Table 3).

#### 3.4.1. MicroScan WalkAway (Beckman Coulter, San Diego, CA, USA)

It uses standard trays that are manually inoculated, and reading is based on fluorometry, with results obtained in 3.5–7H. It is not available on polymyxin B, and essential agreement cannot be evaluated between techniques as the NM44 panel proposes only ≤4 and ≥4* µ*g/ml for colistin. In the recent study of Chew et al., this panel was able to detect all *mcr-1* tested isolates and presented only one VME on 76 Enterobacteriaceae isolates tested. It also evaluated 213 *Acinetobacter* species and presented 99.1 % categorical agreement against the agar dilution method [115].

#### 3.4.2. Vitek 2 (BioMérieux, Marcy l'Étoile, France)

It is a semiautomated system that uses reagent cards containing dehydrated antibiotics and other reagents in a 64-well format. It combines rapid identification and AST using an extrapolated growth algorithm. Various comparative studies performed to evaluate Vitek 2 have returned discrepant results with high rates of VME. In their recent evaluation, Chew et al. [147] demonstrated the efficacy of Vitek 2 in assessing both polymyxin B and colistin MIC with only one VME for each and 96.1% and 93.9% EA, respectively, but it was only able to detect *mcr-1* strains with polymyxin B.

#### 3.4.3. BD Phoenix™ (Becton Dickinson, Le Pont de Claix, France)

It performs identification and AST in parallel in 84-well specific plates. Reading is based on an oxidation-reduction indicator in 6–16 hours. One study showed excellent agreement on 109 *P. aeruginosa* strains, but only colistin-susceptible strains were tested [149]. Vourli et al. [129] have shown concerning results testing *Acinetobacter baumannii* strains with very high VME rates (41.4%) despite the study including 24.8% of colistin-resistant strains. This was explained by the majority of errors occurring near the breakpoint (2 instead of 4* µ*g/ml). Lastly, in the study by Jayol et al. [83], the Phoenix system was able to detect all *mcr*-carrying bacteria, even those with a colistin MIC of 4* µ*g/ml, but the high rates of VMEs obtained prove its inability to assess heteroresistance.

## 4. Genotyping and Molecular Screening

### 4.1. PCR Amplification and Sequencing to Detect Gene Mutations

Molecular biology methods are the most sensitive for determining antibiotic resistance by assessing the presence of resistance genes or mutations conferring resistance. These methods are complementary to the phenotypic techniques and confirm the resistant status of bacterial isolates. The main mutations for Enterobacteriaceae species are located on genes coding the two-component systems PmrA/PmrB and PhoP/PhoQ (Figure 1). Specifically, mutations in the *mgrB* gene—the negative feedback regulator of PhoPQ—notably with the presence of insertional sequences, appeared to be the main resistance mechanism observed in *K. pneumoniae* strains. These colistin resistances are not based on drug-modifying enzymes or the acquisition of a resistance gene which could be easily detected. Screening of potential mutations on these chromosomal genes is done by amplification and sequencing, takes 3 days, and requires that all genes are tested. Sequenced amplicons are then compared by the BLAST tool against the NCBI database to screen possible mutations compared to wild-type genes.

### 4.2. Real-Time qPCR to Detect the Presence of *mcr* Genes

**Figure 2:**
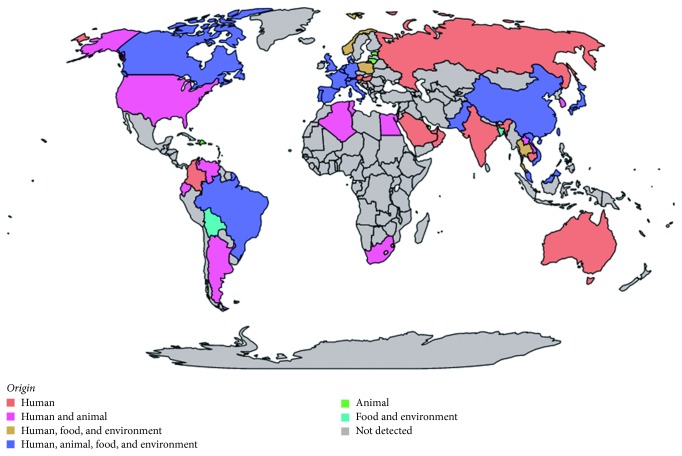
Worldwide dissemination of the *mcr-1* gene. The map was performed with Magrit mapping application (http://magrit.cnrs.fr).

The discovery of the acquired gene *mcr-1* justifies the use of molecular detection with RT-PCR, a rapid quantitative technique to detect the presence of the gene. A systematic screening of the gene in colistin-resistant strains was performed [150] (Figure 2). For such purposes, scientists have used the primers of the original study [6], or have designed their own primers for standard PCR [132, 151–160] or RT-PCR, based on SYBR Green assays [64, 151, 161], TaqMan probe [66, 72, 152, 162], or other FAM-labelled probe [9, 71, 163, 164] or HEX-labelled probe [165] (Table 4).

Xavier et al. designed primers to screen *mcr-2* [7], giving a 567 bp product [166]. Some designed their own primers for standard PCR [167–169], and one study developed a TaqMan assay for qPCR [170]. Interestingly, three studies went further by designing a universal primer to detect both *mcr-1* and *mcr-2* genes by standard PCR [166, 171] and a generic primer and a probe to detect them by qPCR [74], but these have not yet been tested on other *mcr* genes. Lastly, primers were designed for detecting *mcr-3* [10], *mcr-4* [11], and *mcr-5* genes [12] by standard PCR (Table 4). A recent study described a multiplex SYBR Green real-time PCR assay for the simultaneous detection of *mcr-1*, *mcr-2*, and *mcr-3* genes [172]. Finally, a multiplex PCR assay for detection of the five *mcr* genes: *mcr-1, mcr-2, mcr-3, mcr-4,* and *mcr-5*, was developed in order to obtain sequential amplicons with a size difference of 200 bp, allowing their fast and simultaneous detection on agarose gels [173].

### 4.3. PCR to Detect Plasmid Carrying *mcr* Genes

*mcr-1* is a 1626-base pair-long gene located on a 2607 bp common region flanked on both ends by an IS*Apl1* insertion sequence (IS) in some plasmids [174]. This sequence may form a composite transposon that can potentially move as one complete unit [155, 175, 176]. This insertion sequence appears to be a key component of the mobilome, and its presence is not systematic. Furthermore, only the upstream region can contain IS*Apl1* [165]. Li et al. identified the ability of the Tn6330 transposon (IS*Apl1*-*mcr-1*-orf-IS*Apl1*) to generate circular IS*Apl1*-*mcr-1*-orf [177]. Specific primers were developed to screen the upstream presence of this IS transposon by PCR and Sanger sequencing [178–180]. Others have also designed their own system to directly screen on plasmid carrying *mcr*-gene type IncX4 [9, 181], but these methods also exhibit limitations, as a wide distribution has been observed for *mcr-1* among different plasmids (IncI2, IncX4, IncHI2, IncY, IncF, IncP, IncH1, and IncX3), demonstrating the great ability of *mcr* genes to spread.

### 4.4. Microarray

Microarray technology allows scientists to analyze dozens of genes at the same time. The Check-MDR CT103 microarray system (Check-Points, Wageningen, the Netherlands) was developed to screen the presence of extended-spectrum beta-lactamase (ESBL) genes (TEM and SHV) and carbapenemase genes (OXA-48, KPC, NDM…) in the same assay and can assay 24 samples at the same time, with an effective detection in 6.5 hours. Recently, a study evaluated this assay for detecting *mcr* genes: sensitivity and specificity were excellent for *mcr-1* and its variants (from *mcr-1.2* to *mcr-1.7* and *mcr-2* genes), but it was not able to detect the new gene *mcr-3* [182]. *mcr-4* has not been assayed yet.

### 4.5. Loop-Mediated Isothermal Amplification (LAMP)

The Eazyplex SuperBug *mcr-1* kit (Amplex Biosystems Gmbh, Giessen, Germany) was developed to assess the presence of the *mcr-1* gene within 20 minutes [183]. It was effective on 104 microbial strains but needs to be assayed directly on clinical samples. As it was developed before the description of the other *mcr* genes, it can only detect the *mcr-1* gene and the *mcr-1.2* variant.

More recently, another LAMP-based assay was developed to detect *mcr-1* gene and evaluated as a screening tool on 556 multidrug-resistant Enterobacteriaceae [184]. Seven isolates were positive by both standard PCR and LAMP-based assay (6 *E. coli* and 1 *K. pneumoniae*). The results can be assessed by chromogenic visualization. This test constitutes a rapid, specific, and cost-effective tool that exhibits a higher sensitivity than PCR (10-fold). It has to be assayed on clinical samples; as for now, only spiked tools were used.

### 4.6. Novel Approach with Direct Resistome Analysis

Genomic screening is an alternative approach for studying resistance and providing a better understanding of the behavior of bacterial isolates [185]. The development of next-generation sequencing has led to lower costs, reduced screening delay, and increased sequencing speeds combined with updated databases providing access to a large amount of information. The *mcr-1* gene was initially discovered by whole genome sequencing during an active livestock monitoring program in China [6]. A considerable number of retrospective studies analyzing previously recorded genomic sequences have since been carried out, showing the global dispersion of the gene [9, 10, 20, 23, 24, 72, 74, 157, 158, 174, 177, 180, 186–204] (Figure 2).

The technologies used to completely sequence the bacterial genome are Illumina (Illumina Inc., San Diego, CA, USA), which produces short sequences (300 bp) and requires several days, and PacBio RS II (Pacific Biosciences, Menlo Park, CA, USA), which produces a single real-time molecule producing long sequences (60kb) in a few hours [205]. The use of Illumina sequencers is not suitable for covering bacterial genomes with multiple repetitive elements because too many sequence pieces are obtained after assembly, whereas PacBio RS II delivers a single sequence without missing regions [174]. Sekizuka et al. performed a hybrid analysis using the two technologies to analyze three Inc2 plasmids and found that they were highly conserved with the exception of the shufflon region, meaning that the combination of the two methods enables to analyze rearragements in highly recombinant regions [174].

The sequences obtained are assembled, the genome is annotated, and then a mapping is carried out against a reference plasmid, in general pHNSHP45 for *mcr-1* [6]. The aligned sequences are then compared to one of the resistance gene databases: Antibiotic Resistance Gene-Annotation [206], ResFinder [207], Comprehensive Antibiotic Resistance Database [208], and Antibiotic Resistance Genes Database [209]. They could also be compared to the plasmid genome, with GenEpid-J [210] or PlasmidFinder, which enabled the discovery of the *mcr-4* gene [11] and presents the advantage of screening multiple genes and detecting the coexistence of several genes including carbapenemases. Lindsey et al. proposed a whole protocol for plasmid sequencing [211]. More specifically, PointFinder was developed to detect chromosomal point mutations associated with antimicrobial resistance [212].

Whole genome sequencing combined with new bioinformatic tools improves our ability to detect several resistance genes at the same time [186, 205] but presents the same limitations than PCR: new genes are not recognized by these techniques, which require the continuous updating of databases [175, 213] that should be merged into a single reference database [213].

## 5. Conclusion

The recent description of plasmid-mediated colistin-resistant genes has generated concern among the global scientific community about the lack of new antibiotics to treat infections caused by multidrug-resistant pathogens. This concern was raised by the worldwide screening that demonstrated the global spread of bacterial strains harboring the *mcr-1* gene from diverse human and animal origins. Thus, it is necessary to implement an adapted protocol to effectively detect colistin-resistant strains in clinical microbiology laboratories.

**Figure 3:**
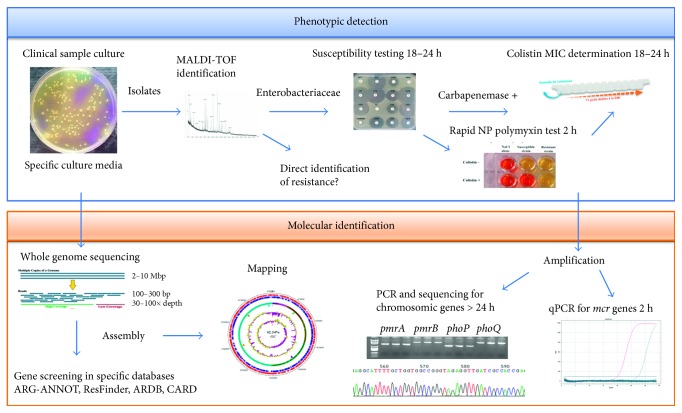
Complementarity of phenotypic and genotypic methods in detection and analysis of colistin-resistant bacteria.

Phenotypic methods indicate to the microbiologist the presence of polymyxin-resistant strains but do not define the mechanism involved and the risk of transmission. Molecular methods are rapid and more sensitive but are specific to the resistance genes examined and faced with the large number of molecular mechanisms conferring resistance to polymyxins, should only be used to screen *mcr* genes in clinical microbiology laboratories. Genomic analysis enables the complete screening of resistance genes in genetically identified bacteria from clinical samples but remains an *in silico* study which enables predictions but not resistance observation, as the presence of a resistance gene in a genome does not mean that the corresponding isolate is resistant, supported by studies that identified polymyxin-susceptible bacteria carrying the *mcr-1* gene [92, 165, 213]. Thus, phenotypic and molecular methods are complementary in detecting colistin-resistant pathogens in order to analyze the behavior of the clinical isolate, and it is important to carry them out in parallel [148] (Figure 3). All these techniques and their detection characteristics are summarized in Table 5.

In conclusion, these new techniques need to be combined for a complete understanding of colistin resistance, in particular for strains carrying *mcr* genes, so clinicians can rapidly adapt treatments or isolate the carrier patient in the hospital.

## Figures and Tables

**Table 1:** Composition of polymyxin-containing agar.

Targeted bacteria	Culture medium	Antibiotics (*µ*g/mL) targeting				References
		Gram-negative strains		Gram-positive strains	Yeast	
		Polymyxins	Others			
*Colistin-resistant Gram-negative strains*	LBJMR^a^	4 (C)		Vancomycin 50		
*VancoR Gram-positive strains*						
*Gram-negative strains*						
Colistin-resistant	SuperPolymyxin	3.5 (C)		Daptomycin 10 BM 65 Éosine 400	5 (AB)	[75]
*Neisseria* sp.	Martin–Lewis agar	7.5 (C)	5 (T)	Vancomycin 4	20 (A)	[34]
	Thayer–Martin agar	7.5 (C)		Vancomycin 3	2.57 (N)	[35]
	MTM^b^ agar	7.5 (C)	5 (T)	Vancomycin 3	2.57 (N)	[36]
	NYC^c^ agar	7.5 (C)	3 (T)	Vancomycin 2	20 (A)	[37]
*Burkholderia cepacia*	Cepacia medium	30 (B)		Ticarcillin 100		[38]
	OFPBL^d^ agar	30 (B)	Bacitracin 3			[39]
	*Burkholderia cepacia* agar	17.8 (B)	5 (GEN)	Ticarcillin 100		[38]
	*Burkholderia cepacia* selective agar	71.4 (B)	10 (GEN)	Vancomycin 2.5		[40]
*Legionella* sp.	BCYE^e^ selective agar with					
	GVPC^f^	9.4 (B)	Glycine 3000	Vancomycin 1	80 (CH)	[41]
	CCVC^g^	16 (C)		Vancomycin 0.5 Cefalotin 4	80 (CH)	[42]
	GPVA^h^	11.9 (B)	Glycine 3000	Vancomycin 1	80 (A)	[43]
	PAV^i^	4.76 (B)		Vancomycin 0.5	80 (A)	[44]
	PAC^j^	9.52 (B)		Cefamandole 2	80 (A)	[45]
	DGVP^k^	8 (B)	Glycine 3000	Vancomycin 1		[46]
*Campylobacter* sp.	*Campylobacter* agar					
	Butzler	0.33 (C)	Bacitracin 338	Novobiocin 5 Cefazolin 15	50 (CH)	[47]
	Skirrow	0.25 (B)	2.5 (T)	Vancomycin 5		[48]
	Blaser–Wang	0.125 (B)	2.5 (T)	Vancomycin 5 Cefalotin 15	2 (AB)	[49]
	Preston	0.125 (B)	5 (T)	Rifampicin 5	50 (CH)	[50]
*Brucella* spp.	*Brucella* selective medium	1 (B)	Bacitracin 500		100 (CH)	[51]
*Vibrio* sp.	CPC^l^	66.34 (C) 11.9 (B)				[52]
*Gram-positive strains*						
*Streptococcus* sp. and Gram-positive strains	ANC^m^	10 (C)	Nalidixic acid 10			[53]
*Listeria monocytogenes*	Oxford medium	20 (C)	Fosfomycin 10	Cefotetan 2 Acriflavine 5	400 (CH)	[54]
	Modified Oxford	10 (C)		Moxalactam 15		[55]
*Listeria* spp.	PALCAM^n^	10 (B)		Ceftazidime 8 Acriflavine 5		[56]
*Bacillus cereus*	MYP^o^	10 (B)				[57]
Mycobacteriaceae	Middlebrook 7H11	25 (B)	20 (T)	Carbenicillin 50	10 (AB)	[58]
*Clostridium perfringens*	SPS^p^ agar	10 (B)	Sulfadiazine 120			[59]
	TSN^q^ agar	20 (B)	Neomycin 50			[60]
	SFP^r^ agar	3.57 (B)	Kanamycin 12			[61]

B: polymyxin B; C: colistin; AB: amphotericin B; A: anisomycin; CH: cycloheximide; MB: methylene blue; N: nystatin; GEN: gentamicin; T: trimethoprim. ^a^LBJMR: Lucie Bardet–Jean-Marc Rolain; ^b^MTM: modified Thayer–Martin; ^c^NYC: New York City; ^d^OFPBL: oxidation/fermentation, polymyxin B, bacitracin, and lactose; ^e^BCYE: buffered charcoal and yeast extract; ^f^GPVC: glycine, polymyxin B, vancomycin, and cycloheximide; ^g^CCVC: cefalotin, colistin, vancomycin, and cycloheximide; ^h^GPVA: glycine, polymyxin B, vancomycin, and anisomycin; ^i^PAV: polymyxin B, anisomycin, and vancomycin; ^j^PAC: polymyxin B, anisomycin, and cefamandole; ^k^DGVP: dyes, glycine, vancomycin, and polymyxin B; ^l^CPC: cellobiose, polymyxin B, and colistin; ^m^CNA: colistin and nalidixic acid; ^n^PALCAM: polymyxin B, acriflavine, lithium, ceftazidime, esculin, and mannitol; ^o^MYP: mannitol, egg yolk, and polymyxin B; ^p^SPS: sulfite, polymyxin B, and sulfadiazine, ^q^TSN: trypticase, sulfite, and neomycin; ^r^SFP: Shahidi-Ferguson perfringens.

**Table 2:** Joint EUCAST-CLSI recommendations on colistin susceptibility testing.

Reference method	Broth microdilution		
Preparation according to ISO 20776-1 standard [102]	(i) Cation-adjusted Mueller-Hinton medium (MH2)		
	(ii) Colistin sulfate		
	(iii) Polystyrene trays without pretreatment		
	(iv) Absence of polysorbate 80 or any surfactant		

MIC breakpoint (*µ*g/ml)	Enterobacteriaceae	*P. aeruginosa*	*Acinetobacter* spp.
EUCAST [100]	*S* ≤ 2 and *R* > 2	*S* ≤ 2 and *R* > 2	*S* ≤ 2 and *R* > 2
CLSI [103]	ECV^*∗*^: WT ≤ 2 and NWT ≥4	*S* ≤ 2 and *R* ≥ 4	*S* ≤ 2 and *R* ≥ 4

Quality control [104] (*µ*g/ml)	*E. coli* ATCC 25922	*P. aeruginosa* ATCC 27853	*E. coli* (*mcr-1*) NCTC 18853^#^
Target	0.5–1	1–2	4
Range	0.25–2	0.5–4	2–8

^*∗*^Epidemiological cutoff values: clinical data and PK/PD are not sufficient to evaluate a clinical breakpoint for the following species: *E. aerogenes*, *E. cloacae*, *E. coli*, *K. pneumoniae*, and *R. ornithinolytica*. WT: wild type; NWT: non-wild type. ^#^Recommended by EUCAST; MIC must be 4 *µ*g/ml and only occasionally 2 or 8 *µ*g/ml.

**Table 3:** Comparison of different colistin susceptibility testing methods to detect colistin resistance in Gram-negative clinical isolates.

Bacterial species	Reference method	MIC breakpoint	MIC range; % resistant	MIC50 (*µ*g/ml)	MIC90 (*µ*g/ml)	Methods	CA ≥ 90%	EA ≥ 90%	ME ≤ 3%	VME ≤3%	MiE	References
42 *A. baumannii* isolates	BMD	*S* ≤ 2 *µ*g/ml	0.5–4 *µ*g/ml	1 *µ*g/ml	2 *µ*g/ml	BMD in glass-coated plates	92.8	100	0	100		[114]
						AD	78.5	92.8	15.4	100		
		*R* > 2 *µ*g/ml	0.07%			E-test	92.8	16.6	0	100		
						Vitek 2^2^ AST-N2812	92.8	61.9	0	100		

353 isolates	BMD	*S* ≤ 2 *µ*g/ml	ND	ND	ND	Sensitest	98.9	96	1.46	0.93		[146]
(83 *mcr-1*)		*R* > 2 *µ*g/ml	38.8%									

219 isolates		*S* ≤ 2 *µ*g/ml	ND	ND	ND	Phoenix 100^3^ NMIC-417	96.8	ND	0.6	10		[146]
		*R* > 2 *µ*g/ml	27.4%									

14 *E. coli* isolates	BMD	*S* ≤ 2 *µ*g/ml	0.25–128 *µ*g/ml; 48%	2	16	Sensititre^1^ SEMPA1	94.7	96	10.2	0		[143]
18 *K. pneumoniae* isolates		*R* > 2 *µ*g/ml				Micronaut-S	89.3	96	15.4	5.6		
21 *P. aeruginosa* isolates						Micronaut-MIC	90.7	99	12.8	5.6		
22 *Acinetobacter* spp. isolates						Etest, Oxoid MH	81.3	71	5.1	33.3		
						Etest, BBL MH	78.7	43	2.6	41.7		
						Etest, MHE	85.3	47	5.1	25		
						MTS, Oxoid MH	78.7	40	0	44.4		
						MTS, BBL MH	76	49	0	50		
						Sensitest	89.3	88	17.9	2.8		
						UMIC	92	82	7.7	8.3		

117 *A. baumannii* isolates	BMD	*S* ≤ 2 *µ*g/ml	≤0.5–≥16 *µ*g/ml; 24.8%	≤0.5	8	Vitek 2 AST-XN05	89.7	88.9	1.1	37.9		[129]
		*R* > 2 *µ*g/ml				Phoenix 100 NMIC/ID-96	88.9	91.5	1.1	41.4		
						AD	87.2	93.2	15.9	3.4		

123 Enterobacteriaceae isolates (14 *mcr-1* and 1 *mcr-2*)	BMD	*S* ≤ 2 *µ*g/ml	0.12–128 *µ*g/ml; 67.5%			Phoenix 100 NMIC-93	91.8	ND	0	12.1		[83]
		*R* > 2 *µ*g/ml				Rapid NP	98.3	NA	2.5	1.2		

15 *Hafnia alvei* isolates	BMD	*S* ≤ 2 *µ*g/ml	0.125–32 *µ*g/ml; 96%	8	16	DD	4	NA	0	100		[79]
10 *Hafnia paralvei* isolates		*R* >2 *µ*g/ml		8	8	Etest	76	32	0	25		
						Phoenix NMIC-93	100	NA	0	0		
						Rapid NP	100	NA	0	0		

76 Enterobacteriaceae isolates (21 *mcr-1*)	BMD	*S* ≤ 2 *µ*g/ml	0.06–>64 *µ*g/ml; 32.9%	0.25	16	Vitek 2 AST N315	88.2	93.9	0	36		[147]
		*R* >2 *µ*g/ml		2 (4)	8 (8)	Sensititre GNX3F	90.1	89.5	11.8	4		
				0.12	0.5	Etest	92.1	75	5.9	12		
						MicroScan^4^ NM44	88.2	NA	15.8	4		

246 isolates (absence of *mcr* genes)	Broth macrodilution	*S* ≤ 2 *µ*g/ml	≤0.5–>8 *µ*g/ml; 12.6%	≤0.5	8	Etest	95.1	92.3	0.4	35.5		[160]
		*R* >2 *µ*g/ml										

41 *K. pneumoniae* isolates	BMD	*S* ≤ 2 *µ*g/ml	2–>128 *µ*g/ml; 95.1%	8	32	BMD-P80	82	95.1	0	18.9	NA	[110]
20 *A. baumannii* isolates		*R* > 2*µ*g/ml		8	32	AD	91.8	55.7	100	3.4		
						Etest	59	50.8	33.3	39.3		
						MTS	67.2	65.6	33.3	41.4		
						Vitek 2 AST EXN8	96.7	70	66.6	0		

290 *A. baumannii* isolates	BMD	*S* ≤ 2 *µ*g/ml	1–128 *µ*g/ml; 9.3%	2	2	DD 10 *µ*g (9–12 mm)	94.8	NA	0	0	5.2	[136]
		*R* > 2 *µ*g/ml				Etest *S* ≤ 2; *R* > 4	94.5	2.1	0	55.5	0	
						Etest *S* ≤ 0.5; *R* > 2	99.3	≡	0	0	5.5	
						Vitek 2 AST-N136	94.1	44.8	0.38	59.2		

213 *Acinetobacter* sp. isolates	AD	*S* ≤ 2 *µ*g/ml	≤0.5–≥32 *µ*g/ml; 6.1%	1	2	Vitek 2 AST-N132	99.1	ND	0	15.4		[115]
		*R* >2 *µ*g/ml				Etest	87.3		1	0		
						MicroScan panel type 42	99.1		12.5	15.4		

60 *P. aeruginosa* isolates	BMD-P80	*S* ≤ 2 *µ*g/ml	≤0.12–≥8 *µ*g/ml; 17.8%			Broth macrodilution	98	83	2.3	0		[128]
20 *K. pneumoniae* isolates		*R* >2 *µ*g/ml			>8	Etest	91	61	4.5	31.6		
27 *A. baumannii* isolates					>8							

11 *A. baumannii* isolates	BMD-P80	*S* ≤ 2 *µ*g/ml	≤0.12–≥8 *µ*g/ml; 20%		>8	BMD	88	34^*∗*^	12.5	10		[128]
15 *K. pneumoniae* isolates		*R* >2 *µ*g/ml			>8	AD	94	80	7.5	0		
24 *P. aeruginosa* isolates						Sensititre GNXF	96	62^*∗*^	5	0		

11 *A. baumannii* isolates	BMD-P80	*S* ≤ 2 *µ*g/ml	≤0.12–≥8 *µ*g/ml; 30%			Etest, BBL MH	78	46	5.7	47		[128]
15 *K. pneumoniae* isolates		*R* > 2 *µ*g/ml				Etest, Hardy MH	78	64	2.8	53		
24 *P. aeruginosa* isolates					>8	Etest, Remel MH	84	68	2.8	47		

109 *P. aeruginosa* isolates	BMD	*S* ≤ 2 *µ*g/ml	0%			Phoenix NMIC/ID-76	100	99.1	0	0		[149]
		*R* >2 *µ*g/ml										

63 *E. coli* isolates	BMD	*S* ≤ 2 *µ*g/ml	0.12–16 *µ*g/ml; 18.6%		1	BMD-P80	99.2	41.3	0	43.5		[112]
61 *K. pneumoniae* isolates		*R* >2 *µ*g/ml			0.5							
60 *Acinetobacter* spp. isolates					2							
63 *P. aeruginosa* isolates					2							

200 Enterobacteriaceae isolates	AD	*S* ≤ 2 *µ*g/ml	0.128–>128 *µ*g/ml; 28.5%			DD 50 *µ*g; *R* < 15 mm	96.5	NA	0	12.3		[116]
82 *K. pneumoniae* isolates		*R* > 2 *µ*g/ml		0.5	128	DD 10 *µ*g; *R* ≤ 8; *S* ≥ 11 mm	93	NA	0	8.8	4.5	
51 *E. coli* isolates				0.5	0.5	DD 10 *µ*g; *R* ≤ 11; *S* ≥ 14 mm	99.5	NA	0	1.7	26.5	
67 E. cloacae isolates				0.5	2	Etest	100	52	0	0	0	

25 *P. aeruginosa* isolates	AD	*S* ≤ 2 *µ*g/ml	0.25–≥256 *µ*g/ml; 57.1%	2	>256	BMD	81.1	40.5	0	25	5.4	[120]
12 *S. maltophilia* isolates		*R* > 4 *µ*g/ml		>256	>256	Etest	74.3	56.7	0	35	11.4	
						DD 10 *µ*g; *R* ≤ 10; *S* ≥ 11 mm	82.8	NA	0	35	2.9	

157 *E. coli* isolates	AD	*S* ≤ 2 *µ*g/ml	0.25–32 *µ*g/ml; 9.6%	0.5	2	DD 150 *µ*g; *R* < 16; *S* ≥ 20 mm	46.5	NA	1.4	20	49.7	[123]
		*R* >4 *µ*g/ml				DD 10 *µ*g; 2 + 18H^*∗*^ (10–15)	96.8	NA	0.7	13.3	1.9	
						Etest	96.8	81.5	0.7	0	0.6	

78 *P. aeruginosa* isolates	BMD	*S* ≤ 2 *µ*g/ml	<0.25–2 *µ*g/ml; 0	1	1	Etest	100	79.5	0	0	6.4	[135]
		*R* > 4 *µ*g/ml				DD 10 *µ*g	100	NA				

100 *A. baumannii* isolates	Phoenix	*S* ≤ 2 *µ*g/ml		0.5	0.5	DD 10 *µ*g; *R* ≤ 8; *S* ≥ 11 mm	100	NA	0	0		[137]
		*R* > 4 *µ*g/ml				Etest	100	NA	0	0		

154 *Acinetobacter* spp. isolates	AD	*S* ≤ 2 *µ*g/ml	≤0.064–≥32; 11.7%	NA	NA	Etest	98.7	88	0.7	5.6		[124]
		*R* ≥ 4 *µ*g/ml										

170 Gram-negative isolates	AD	*S* ≤ 4 *µ*g/ml	0.25–128; 31.2%			Etest	100	91.2	0	0		[126]
		*R* > 4 *µ*g/ml										

102 Gram-negative isolates	BMD	*S* ≤ 2 *µ*g/ml	<0.5–>64 *µ*g/ml; 50%	NA	NA	AD, Oxoid MH	ND	96.8				[31]
		*R* > 4 *µ*g/ml				AD, Oxoid Iso-Sensitest		97.9				
						Etest, MH		72.6				
						Etest, ISO		64.2				
						Vitek 2 AST N038		93.1				
						DD 10 *µ*g; *R* ≤ 10; *S* ≥ 13 mm		NA				

44 *Acinetobacter* spp. isolates	AD	*S* ≤ 2 *µ*g/ml	1-2 *µ*g/ml; 0	1	2	Vitek 2 AST-N032	100	ND	0	NA	NA	[118]
		*R* > 2 *µ*g/ml										

172 Gram-negative isolates	AD	*S* ≤ 2 *µ*g/ml	0.5–64; 31.4%			Vitek 22 AST-N032 (*n* = 32)	82	75.2	0	57.4		[32]
		*R* > 2 *µ*g/ml				Etest (*n* = 137)	86.6	75.0	6.8	27.8		

115 *A. baumannii* isolates	BMD	*S* ≤ 2 *µ*g/ml	≤0.06–512 *µ*g/ml; 19.1%	≤0.06	32	Etest	98.2	16.5	0	1.7		[138]
		*R* > 2 *µ*g/ml										

501 *P. aeruginosa* isolates (401 CF)	AD	*S* ≤ 4 *µ*g/ml	≤0.5–≥16 *µ*g/ml; 17.8%	2	4	BMD 24 h	96		1.2	26.5		[121]
50 *A. xylosoxidans* isolates		*R* >4 *µ*g/ml		4	≥16	BMD 48 h	93.6		3.9	18.0		
50 *S. maltophilia* isolates				8	≥16							

70 *S. maltophilia*	AD	*S* ≤ 2 *µ*g/ml	0.12–32 *µ*g/ml; 24.3%	2	4	DD 10 *µ*g; *R* ≤ 8; *S *≥ 11 mm	71.2	NA	0	93.7	5.7	[125]
		*R* > 2 *µ*g/ml				Etest	86.4	96.7	5.6	37.5	NA	

200 Gram-negative isolates	BMD	*S* ≤ 2 *µ*g/ml	≤1–>128 *µ*g/ml; 15%			DD 10 *µ*g; *R* ≤ 10; *S* ≥ 14 mm	94	NA	0	21.8	1.5	[127]
(i) 60 *A. baumannii* isolates		*R* > 2 *µ*g/ml		≤1	2	DD 10 *µ*g; *R* ≤ 8; *S* ≥ 11 mm	95		0	31.2	1	
(ii) 80 *P. aeruginosa* isolates				≤1	≤1							
(iii) 12 *S. maltophilia* isolates				≤1	32							

35 representatives	BMD	*S* ≤ 2 *µ*g/ml	≤1–≥128 *µ*g/ml; 40%			AD	97.1	91.4	47.6	0		[127]
		*R* > 2 *µ*g/ml										

CA: categorical agreement; EA: essential agreement; VME: very major error; ME: major error; MiE: minor error; AD: agar dilution; BMD: broth microdilution; DD: disk diffusion; MH: Mueller-Hinton. Italic values indicate the number of errors and not the percentage when a too few number of strains tested, where *R* for VME or *S* for ME. ^1^Sensititre panels: ≤0.25–>4 *µ*g/ml except for SEMPA1. ^2^Vitek 2 reagent cards: ≤0.5–≥16 *µ*g/ml except for AST-N038 (≤2, 4, and >4 *µ*g/ml) and AST-N032 (1–4 *µ*g/ml). ^3^Phoenix 100 cards: ≤1–>4 *µ*g/ml. ^4^MicroScan-dried Gram-negative breakpoint combo panel type 42 ≤2 and >4 *µ*g/ml. ^*∗*^Prediffusion test: discs were removed after 2 h of incubation.

**Table 4:** List of primers designed to detect *mcr* genes by PCR.

Targeted genes	Analyze	Method	Primer sequences	Cycle (nb: steps)	Product (bp)	Study
*mcr*-1	Original study	Std	CLR F: 5′-CGGTCAGTCCGTTTGTTC-3′	25: 94°C, 30 s; 58°C, 90 s; 72°C, 60 s	309	[6]
			CLR R: 5′-CTTGGTCGGTCTGTAGGG-3′			
	105 colistin-resistant strains	Std	*mcr*-1_F: 5′-TGTGGTACCGACGCTCGGTCAG-3′			[152]
			*mcr*-1_R: 5′-TCAGCGGATGAATGCGGTGC-3′			
	45 colistin-resistant strains in 2 spiked stools	HotStarTaqMasterMix	*mcr*-1_s: 5′-ATGGCACGGTCTATGATA-3′	45: 95°C, 30 s; 55°C, 30 s; 72°C, 30 s	155	[160]
		*Mcr*-1_FAM-BHQ: 5′-CTACAGACCGACCAAGCCGA-3′	*mcr*-1_as: 5′-CGGATAATCCACCTTAACA-3′			
	In silico study	Pr 5 = -HEX-C	*mcr*-1-286F: 5′-ACTTATGGCACGGTCTATGA-3′	40: 95°C, 10 s; −56°C, 40 s		[162]
		CAAGCCGA-ZEN-GACCAAGGATC-3IABkFQ-3	*mcr*-1-401R: 5′-ACACCCAAACCAATGATACG-3′			
	20 strains in 3 spiked stools	SYBR Green	*mcr*-1-qF1: 5′-ACACTTATGGCACGGTCTATG-3′	40: 95°C, 3 s; 60°C, 20 s; 72°C, 7 s	120	[148]
			*mcr*-1-qR1: 5′-GCACACCCAAACCAATGATAC-3′			
			*mcr*-1-qF2: 5′-TGGCGTTCAGCAGTCATTAT-3′			[165]
			*mcr*-1-qR2: 5′-AGCTTACCCACCGAGTAGAT-3′			
	2046 strains	Std	*mcr*-1-F: 5′-ATGATGCAGCATACTTCTGTGTG-3′		1646	[148]
			*mcr*-1-R: 5′-TCAGCGGATGAATGCGGTGC-3′			
	Wastewater samples	SYBR Green	*mcr*-1-F1: 5′-TGTTCTTGTGGCGAGTGTTG-3′	40: 95°C, 15 s; 60°C, 30 s		[158]
			*mcr*-1-R1: 5′-CGCGCCCATGATTAATAGCA-3′			
	78 stool	SYBR Green	*mcr*-1-FW: 5′-ACGCCATCTGCAACACCAA-3′	30/40: 95°C, 15 s; −63°C, 10 s; −72°C, 10 s	59	[61]
			*mcr*-1-RV: 5′-GCCAACGAGCATACCGACAT-3′			
	100 strains: 18 colistin-resistant strains in 833 faecal samples	TaqMan probe: 6 FAM–GACCGCGACCGCCAATCTTACC-TAMRA	F1: GCAGCATACTTCTGTGTGGTAC	35: 95°C, 30 s; −60°C, 1 min	145	[149]
			R1: ACAAAGCCGAGATTGTCCGCG			
		Std	F1: GCAGCATACTTCTGTGTGGTAC		554	
			R3: TATGCACGCGAAAGAAACTGGC			
	1495 *E. coli* strains and 571 KP strains	Std	*Mcr*-1-forward: 5ʹ-GCTCGGTCAGTCCGTTTG-3ʹ			[150]
			*Mcr*-1-reverse: 5ʹ-GAATGCGGTGCGGTCTTT-3′			
	51 strains	FastStart Universal Probe Master kit	M-F: CATCGCGGACAATCTCGG	40: 95°C, 15 s; −60°C, 1 min	116	[161]
	18 samples	FAM-AACAGCGTGGTGATCAGTAGCAT-BHQ	M-R: AAATCAACACAGGCTTTAGCAC			
	241 isolates	Std	*MCR*-1-F2: 5′-CTCATGATGCAGCATACTTC-3′		Entire gene	[151]
			*MCR*-1-R2: 5′-CGAATGGAGTGTGCGGTG-3′			
	Clinical *E. coli* isolates	TaqMan Fast Advanced Master Mix	*MCR*-1F: 5′-CATCGCTCAAAGTATCCAGTGG-3′			[69]
		5′-Cy5-TGCAGACGCACAGCAATGCCTATGAT-TAO-3′	*MCR*-1R: 5′-CCATGTAGATAGACACCGTTCTCAC-3′			
	10,609 *E. coli* isolates (505R)	TaqMan RT-*mcr*-1_Probe	RT-*mcr*-1_F: TGGCGTTCAGCAGTCATTAT	30°C–95°C, 15 s; −60°C, 1 min		[159]
		Cy5-AGTTTCTTTCGCGTGCATAAGCCG-BBQ-650	RT-*mcr*-1_R: AGCTTACCCACCGAGTAGAT			
	62 isolates	*MCR*1_22,763_Pb1 FAM-TGGTCTCGG/ZEN/CTTGGTCGGTCTGTAGGGC-3IABkFQ	*MCR*1_22,697_F1: 5′-CACTTATGGCACGGTCTATGA-3′			[68]
			*MCR*1_22,810_R1: 5′-CCCAAACCAATGATACGCAT-3′			
	31 colistin-resistant isolates	Std	*mcr*-1_F: 5′-ATGATGCAGCATACTTCTGTGTGG-3′			[157]
			*mcr*- 1_R: 5′-GTGCGGTCTTTGACTTTGTCC-3′			
	122 faecal samples	TaqMan probe: 5′-TTGACCGCGACCGCCAATCTTA-3′ FAM	[6]	45: 15 s, 95°C; −1 min, −60°C	309	[63]
	48 *E. coli* and 27 KP strains		*mcr*-1-F1: 5′-ATGATGCAGCATACTTCTGTG-3′			[153, 155]
			*mcr*-1-R1: 5′-TCAGCGGATGAATGCGGTG-3′			
			CLR5-F1: 5′-ATGATGCAGCATACTTCTGTGTGG-3′			[156]
			CLR5-R1: 5′-TCAGCGGATGAATGCGGTGC-3′			
			CLR5-F: 5′-CGGTCAGTCCGTTTGTTC-3′			[176]
			*Mcr*1-Rv2: 5′-CCAGCGTATCCAGCACATTT-3′			

*mcr*-2	136 colistin-resistant isolates	Std	*MCR*2-IF: 5′-TGTTGCTTGTGCCGATTGGA-3′	33: 95°C, 3 min; 65°C, 30 s; 72°C, 1 min	567	[7]
	31 coli-resistant isolates		*MCR*2-IR: 5′-AGATGGTATTGTTGGTTGCTG-3′			[157]
	1200 isolates					[163]
	6 isolates	Std	*MCR*-2-F(EcoRI): 5′-AACCGAATTCATGACATCACATCACTCTTG-3′			[164]
			*MCR*-2-R (SalI): 5′-CCGGTCGACTTACTGGATAAATGCCGCGC-3′			
	2396 strains	Std	*Mcr*-2 full Fw: 5′-ATGACATCACATCACTCTTGG-3′	34: 95°C, 1 min; 52°C, 30 s; 72°C, 1 min		[165]
	1144 samples		*Mcr*-2 full Rv: 5′-TTACTGGATAAATGCCGCGC-3′			[65, 166]
	436 cultures	TaqMan *mcr*-2_Probe	*Mcr*-2_fwd: TTGTCGTGCTGTTATCCTATCG	30: 95°C, 15 s; −60°C, 1 min		[167]
		ROX-ACTGATTATGGGTGCGGTGACGAG-BHQ-2	*Mcr*-2_rev: CCGTGCCATAAGTATCGGTAAA			

*mcr*-1 and *mcr*-2	1200 isolates	Std	*mcr*1-2 universal F: ACTTATGGCACGGTCTATGATAC	30: 94°C, 30 s; 58°C, 30 s; 72°C, 2 min	1311	[163]
			*mcr*1-2 universal R: CCGCGGTGACATCAAACA			
		Std	*MCR*-1/2-Fw: 5′-TAT CGC TAT GTG CTA AAG CC-3′		715 bp	[168]
			*MCR*-1/2-Rv: 5′-TCT TGG TAT TTG GCG GTA TC-3′			
	621 faecal samples	*Mcr*-generic probe TATCACGCCACAAGATAC	*Mcr*-generic fw: GCCAAATACCAAGAAAATG		98 bp	[71]
			*Mcr*-generic rev: TTATCCATCACGCCTTTT			

*mcr*-3	580 *E. coli* strains	Std	*MCR*3-F: 5′-TTGGCACTGTATTTTGCATTT-3′	30: 95°C, 30 s; 50°C, 30 s; 72°C, 45 s	542	[10]
			*MCR*3-R: 5′-TTAACGAAATTGGCTGGAACA-3′			

*mcr*-1, *mcr*-2, and *mcr*-3	25 isolates: 17 *mcr*-1 and 8 *mcr*-3 20 samples	SYBR Green	*mcr*1-qf: AAAGACGCGGTACAAGCAAC *MCR*-1	40: 95°C, 30 s; 60°C, 30 s; 72°C, 30 s	213	[169]
			*mcr*1-qr: GCTGAACATACACGGCACAG		92	
			*mcr*2-qf: CGACCAAGCCGAGTCTAAGG *MCR*-2		169	
			*mcr*2-qr: CAACTGCGACCAACACACTT			
			*mcr*3-qf: ACCTCCAGCGTGAGATTGTTCCA *MCR*-3			
			*mcr*3-qr: GCGGTTTCACCAACGACCAGAA			

*mcr*-4	125 isolates	Std	*Mcr*-4 FW: ATTGGGATAGTCGCCTTTTT		487	[11]
			*Mcr*-4 RV: TTACAGCCAGAATCATTATCA			

*mcr*-5	12 Salmonella paratyphi B isolates	Std	*MCR*5_fw: 5′-ATGCGGTTGTCTGCATTTATC-30′	30: 95°C, 30 s; 50°C, 95 s; 72°C, 95 s	1644	[12]
			*MCR*5_rev: 5′-TCATTGTGGTTGTCCTTTTCTG-3′			

*mcr*-1, *mcr*-2, *mcr*-3, *mcr*-4 and *mcr*-5	49 *E. coli* and Salmonella isolates	Std	*mcr*1_fw: AGTCCGTTTGTTCTTGTGGC	25: 94°C, 30s; 58°C, 90s; 72°C, 60s	320	[170]
			*mcr*1_rev: AGATCCTTGGTCTCGGCTTG			
			*mcr*2_fw: CAAGTGTGTTGGTCGCAGTT		715	
			*mcr*2_rev: TCTAGCCCGACAAGCATACC			
			*mcr*3_fw: AAATAAAAATTGTTCCGCTTATG		929	
			*mcr*3_rev: AATGGAGATCCCCGTTTTT			
			*mcr*4_fw: TCACTTTCATCACTGCGTTG		1116	
			*mcr*4_rev: TTGGTCCATGACTACCAATG			
			*mcr*5_fw: ATGCGGTTGTCTGCATTTATC		1644	
			*mcr*5_rev: TCATTGTGGTTGTCCTTTTCTG			

Std: standard; KP: K. pneumoniae.

**Table 5:** Comparison of detection methods for polymyxin resistance.

Method	Principle	Time	Manual (M); automated (A)	Detection			
				ColR	*mcr*	HR	MIC
Phenotypic							
Selective agar	Selective growth	18 h	M	+	−	+	−
Rapid polymyxin NP	pH change	4 h	M	+	−	−	−
Micromax	Cell wall lysis detection by fluorescence	3 h	M/A	+	−	−	−
MALDI-TOF MS	Specific peak detection	1 h	A	+	+	−	−
*MCR-1* inhibition	Chelation with	18 h	M	+	+	±	+
Colistin MAC	Dipicolinic acid						
EDTA assays	EDTA						

AST							
BMD (UMIC, Micronaut-MIC, Sensitest, Micronaut-S, and Sensititre)	Growth inhibition	18 h	M/A	+	−	±	+
Agar diffusion	Measure of growth inhibition zone	18 h	M				
Disk diffusion				−	−	+	−
Gradient strip				−	−	+	+
ColiSpot				+	−	ND	−
Agar dilution	Growth inhibition	18 h	M	−	−	+	+
Automatized system	Growth detection						
MicroScan	Fluorimetry	3.5–7 h	A	+	−	−	−
Vitek 2	Algorithm	4–10 h	A	+	−	−	−
Phoenix	Oxidoreduction	6–16 h	A	+	−	−	−

Genotypic							
Standard PCR	Amplification	3 h	A	+	+	−	−
RT-PCR	Amplification	1 h	A	+	+	−	−
LAMP (Eazyplex, etc.)	Amplification	20 min	A	+	−	−	−
Microarray	DNA hybridization	6.5 h	A	+	−	−	−
NGS	Whole-genome sequencing						
Illumina		4–56 h	A	+	+	−	−
PacBio RS II		0.5–3 h	A	+	+	−	−

ColR: colistin resistance; HR: heteroresistance; +: yes; −: no; ±: sometimes.

## Supplementary Materials

Supplementary Materials Table S1: home-made polymyxin-containing agar. Table S2: comparison of different polymyxin B susceptibility testing methods to detect polymyxin B resistance in Gram-negative clinical isolates. Table S3: other comparative AST methods.
