# Corrigendum to “Development of New Tools to Detect Colistin-Resistance among Enterobacteriaceae Strains”

**DOI:** 10.1155/2019/9083980

**Published:** 2019-05-07

**Authors:** Lucie Bardet, Jean-Marc Rolain

**Affiliations:** Aix-Marseille Université, IRD, AP-HM, MEPHI, IHU-Méditerranée Infection, Marseille, France

In the article titled “Development of New Tools to Detect Colistin-Resistance among Enterobacteriaceae Strains” [1], there was an error in the text of the Selective Culture Media section, where the sentence “The chromogenic and nonselective CHROMagar Orientation medium (Biomérieux, Marcy l'Étoile, France) was also used with 4 mg/ml of colistin” should be corrected to “The chromogenic and nonselective CHROMagar™ Orientation Medium (CHROMagar, Paris, France) was also used with 4 mg/ml of colistin.”
