# Supplementary material for: Development of New Tools to Detect Colistin-Resistance among Enterobacteriaceae Strains
Source: Can J Infect Dis Med Microbiol. 2018 Dec 5;2018:3095249. doi: 10.1155/2018/3095249 (PMC6305056; doi:10.1155/2018/3095249)
Supplement: Supplementary Materials — Table S1: home-made polymyxin-containing agar. Table S2: comparison of different polymyxin B susceptibility testing methods to detect polymyxin B resistance in Gram-negative clinical isolates. Table S3: other comparative AST methods. [file 3095249.f1.pdf]

Supplementary data

Table S1. Home-made polymyxin containing agar

Table S2. Comparison of different polymyxin B susceptibility testing methods to detect polymyxin B resistance in Gram-negative clinical isolates.

Table S3. Other comparative AST methods.

Table S1. Home-made polymyxin containing agar. VAN: vancomycin, CTX: cefotaxime; AB: amphotericin B

Used on:	Agar	Colistin (µg/mL)	Other antimicrobial drugs (µg/mL):	Rreference
Isolates	MH	2		[9]
Cecal samples	1-BPW<br>2-MacConkey	2<br>2		[26]
isolates	MacConkey	2		[27]
Stool samples	1-LB broth<br>2-MacConkey agar<br>Or CHROMagarTM Orientation Medium	2<br>±4<br>4	 <br>8 VAN ± 2 CTX	[28]
Samples	MacConkey	2		[29]
Faecal samples	LB	2	50 VAN	[30]
Faecal samples	LB	4	5 AB	[31]
Stool samples	1-EE<br>2-LB	 4	 10 mg/L VAN<br>5 mg/L AB	[32]
Faecal samples	1- EE broth<br>2-MacConkey	2<br><br>4		[33]
Isolate	COS	2		[34]

Table S2. Comparison of different polymyxin B susceptibility testing methods to detect polymyxin B resistance in Gram-negative clinical isolates.

Bacterial species	Reference method	MIC breakpoint	MIC range<br>% resistant	MIC50	MIC90	Methods	CA<br>≥90%	EA<br>≥90%	ME<br>≤1.5%	VME<br>≤1.5%	MiE	Reference
76 *Enterobacteriaceae*	BMD	S≤2 µg/mL	0.06->64	4	8	Vitek2	*94.7*	96.1	6.1	3.7		[105]
18 *E.coli (13 mcr-1)*		R≥4 µg/mL	35.5%	0.12	0.5	Sensititre	*94.7*	96.1	6.1	3.7		
31 K. pneumoniae 2 mcr-1						Etest	*89.5*	48.7	1.9	26.1		
1 E. aerogenes mcr-1												
70 S. maltophila 15R	AD	S≤2 µg/mL	0.25-16	2	4	DD 300 U	72.8	NA	0	70.6	8.6	[86]
		R≥4 µg/mL	22.7%			Etest	77.3	89.4	13.2	47.1	NA	
200 Gram-negative including:	BMD	S≤2 µg/mL		≤1[a]	2[a]	DD 10 µg[1]	94	NA	0	37.5	0	[88]
60 *A. baumannii*		R≥4 µg/mL	16%	≤1[b]	2[b]	DD 10 µg[2]	94		0	37.5	0	
80 *P. aeruginosa*				2[c]	2[c]							
c. 12 *S.maltophila*												
35 representatives			≤1-≥128<br>40%			AD	97.1	91.4	47.6	0		
78 *P. aeruginosa*	BMD	S≤2 µg/mL	<0.25-8	0.5	1	Etest	90	33		*1.*	48.7	[95]
		I = 4 µg/mL	0.9%			DD	97.3	NA	11.5			
		R≥8 µg/mL										
281 *Enterobacteriaceae*	BMD	S≤2 µg/mL				Etest	49.8	40.9	0	12.5		[78]
		R≥4 µg/mL	8.5%			AD	38.1	50.2	2.7	8.3		
						DD 300U	98.9	NA	0	12.5		
48 K. pneumoniae	BMD	S≤2 µg/mL		≤0.25	8	Etest	75	18.7	26.8	14.3	NA	[94]
		R≥4 µg/mL	14.6%									
63 *E. coli*	BMD	S≤2 µg/mL	0.12-16		2	BMD-P80	97.6	21	0	12.5		[74]
61 *K. pneumoniae*		R≥4 µg/mL	19.4%		1							
60 *Acinetobacter spp.*					2							
63 *P. aeruginosa*					2							
501 *P. aeruginosa* (401 CF)	AD	S≤4 µg/mL	≤0.5-≥16 µg/mL	2	2	BMD 24h	96		22.8	1.7		[82]
50 *A. xylosoxidans*		R≥8 µg/mL	11%	4	8	BMD 48h	93.6		8.4	3.6		
50 *S. maltophila*				4	16							
10 *P. aeruginosa*	BMD	10	≤0.25-4	0.5	1	Etest MH :						[99]
10 *A. baumannii*				0.5	4	Difco	100	100	0	0	0	
						Merck	95	80	0	*1*	0	
						Oxoid	80	80	0	0	*2*	
						HiMedia	90	90	0	0	*4*	

CA: Categorical agreement, EA: Essential agreement, VME: very major error, ME: major error, MiE: minor error, AD: Agar Dilution, BMD: Broth Microdilution, DD: disk diffusion, MH: Mueller Hinton, ISO: ISO Sensitest agar. Italic values indicate number of error and not the percentage for VME or ME when a too few number of resistant susceptible strains were tested respectively.

Table S3. Other comparative AST methods.

Bacterial species	Reference method	MIC breakpoint	MIC range % resistant	MIC50	MIC90	Methods	CA ≥90%	EA ≥90%	ME ≤1.5%	VME ≤1.5%	MiE	Reference
101 KP	Etest	S≤2 µg/mL<br>R≥4 µg/mL	0.25-48<br>16.8%	0.75	16	Microscan	76.3	88.1	11.9	11.8		[93]
146 isolates<br>*56 P. aeruginosa*<br>*29 Acinetobacter spp.*<br>*61 Enterobacteriaceae*	NA					BMD<br>V-bottom<br>+-P80<br>U bottom<br>+ P-80						[75]
169 *E. coli*<br>157 *K. pneumoniae*<br>163 *P. aeruginosa*	BMD					BMD-P80						[73]
778 Gram-negative<br>226 *A. baumanni*<br>124*P.aeruginosa*<br>250 *K. pneumoniae*<br>102 *E. coli*<br>60 *Enterobacter spp.*<br>36 *S. maltophila*	Etest	S≤2 µg/mL<br>I = 4 µg/mL<br>R≥8 µg/mL	≤0.5->16<br>13.1%	 0.5<br>1<br>0.5<br>0.5<br>0.5<br>8	 0.5<br>2<br>16<br>0.5<br>16<br>128	DD 10 µg	87.8	NA	0	0.8	12.1	[102]
200 Gram-negative including	Etest					DD E<br>DD B	75.8<br>78.8	NA<br>NA	0<br>0	75<br>47.1	5.7<br>8.6	[86]
228 isolates:<br>61 *Acinetobacter spp*<br>43 *Enterobacter spp*<br>26 *E. coli*<br>42 *K. pneumoniae*<br>56 *P. aeruginosa*	AD	S≤2 µg/mL<br>R≥4 µg/mL<br><br>[3]S≤4 µg/mL<br>R≥8 µg/mL	0.5->128<br>µg/mL<br>11.8%	 1<br>1<br>2<br>1<br>2	 2<br>≥128<br>2<br>2<br>4	DD MH-<br>10µg[11]S≤8-<br>R>11mm<br>DD MH-<br>10µg[22]S≤10-<br>R>14mm<br>DD ISO-<br>25µg[33]*R≤14mm*<br>*P. aeruginosa*<br>*R*≤13*mm*<br>DD MH-50<br>µg[44]R≤14mm		ND		81.5<br>44.4[#]<br>78.6<br>88.9	 70.6	[80]
246 isolates<br>0 mcr 1 2	MacroD<br>Colistin	S≤2 µg/mL<br>R≥4 µg/mL	≤0.5-8 µg/mL<br>11.8%			Etest<br>Polymyxin B	97.5	96.7	0.5	17.2		[121]
